# Dimorphic Regulation of the *MafB* Gene by Sex Steroids in Hamsters, *Mesocricetus auratus*

**DOI:** 10.3390/ani14121728

**Published:** 2024-06-07

**Authors:** Luis Ramos

**Affiliations:** Department of Reproductive Biology, Instituto Nacional de Ciencias Médicas y Nutrición Salvador Zubirán, México City 14080, Mexico; luis.ramost@incmnsz.mx; Tel.: +52-55-5487-0900

**Keywords:** Harderian gland, macrophages, sexual dimorphism, sex steroids, MafB

## Abstract

**Simple Summary:**

MafB protein is indispensable for fetal testis morphogenesis, and it is important for the differentiation of immune cells such as macrophages. However, our understanding of MafB in rodents remains limited. In this study, MafB cDNA was cloned, and MafB expression was assessed in hamsters (*Mesocricetus auratus*), including gene regulation mechanisms by sex steroids in endocrine and non-endocrine tissues. Furthermore, bioinformatic tools were used to evaluate molecular characterization, three-dimensional structure, and phylogenetic relationships. The study results suggest a possible participation of MafB in the differentiation of Harderian gland (HG) macrophages and pancreatic β cells.

**Abstract:**

MafB is a transcription factor that regulates macrophage differentiation. Macrophages are a traditional feature of the hamster Harderian gland (HG); however, studies pertaining to MafB expression in the HG are scant. Here, the full-length cDNA of the *MafB* gene in hamsters was cloned and sequenced. Molecular characterization revealed that MafB encodes a protein containing 323 amino acids with a DNA-binding domain, a transactivation domain, and a leucine zipper domain. qPCR assays indicated that MafB was expressed in different tissues of both sexes. The highest relative expression levels in endocrine tissues were identified in the pancreas. Gonadectomy in male hamsters was associated with significantly higher mRNA levels in the HG; replacement with dihydrotestosterone restored mRNA expression. The HG in male hamsters contained twofold more MafB mRNA than the HG of female hamsters. Adrenals revealed similar mRNA relative expression levels during the estrous cycle. The estrous phase was associated with higher mRNA levels in the ovary. A significantly up-regulated expression and sexual dimorphism of MafB was found in the pancreas. Therefore, MafB in the HG may play an active role in the macrophage differentiation required for phagocytosis activity and intraocular repair. Additionally, sex steroids appear to strongly influence the MafB expression in the HG and pancreas. These studies highlight the probable biological importance of MafB in immunological defense and pancreatic β cell regulation.

## 1. Introduction

The musculoaponeurotic fibrosarcoma oncogene (Maf) family of proteins are a subgroup of basic-region leucine zipper (bZIP) transcription factors that recognize a long palindromic DNA sequence [TGCTGAC(G)TCAGCA] known as the Maf recognition element (MARE) [1]. Maf protein B (MafB) consists of NH_2_-terminal activation or an acidic domain (rich in proline, serine, and threonine residues), a DNA-binding domain, and a bZIP domain in its COOH-terminal region that is necessary for homo- or hetero-dimerization via its leucine-repeat structure [2,3]. Interestingly, human and mouse *MafB* genes contain no introns [4,5].

In mice, MafB is dispensable for fetal testis morphogenesis and the maintenance of spermatogenesis [6]. MafB is also a transcriptional regulator required for islet α and β cell differentiation [7]. It is furthermore expressed in all developing insulin- and glucagon-producing cells and in a restricted fashion in mature α and β cells [8]. Likewise, MafB is expressed in podocytes and osteoclasts and plays a key role in their differentiation and development [9,10]. In chickens, MafB induction is a specific and essential determinant of the monocytic program in hematopoietic cells, and it is important for macrophage differentiation [11]. Recent studies of *MAFB* gene mutations have demonstrated an association with multicentric carpotarsal osteolysis (MIM number 166300) and Duane retraction syndrome (MIM number 617041) with focal segmental glomerulosclerosis [12,13]. Previous reports have shown that MafB is specifically upregulated in infiltrating macrophages at the site of bacterial infections from peripheral blood [14,15]. In mice, MafB expression is prominent in the mesenchyme of the male genital tubercle (GT), the anlage of external genitalia. MafB expression is downregulated in the male GTs of androgen receptor (AR) knockout mice, indicating that AR signaling is necessary for its expression, as it drives the masculinization of embryonic urethral formation in an androgen-dependent manner. These attributes suggest that MafB is a crucial mediator of urethral masculinization and is a possible new candidate gene for hypospadias [16]. Interestingly, molecular studies have revealed that the expression of the *MAFB* gene and protein in the foreskin of children with hypospadias is lower than those in unaffected children [17]. However, preliminary molecular genetic analyses of patients with hypospadias have reported the absence of *MAFB* gene mutations; this finding suggests that MAFB might play only a limited role in the formation of the human male urethra [18]. Follow-up studies pertaining to the promoter region of the *MAFB* gene could contribute to elucidating the transcriptional regulation mechanisms associated with hypospadias.

The Harderian gland (HG) is an exocrine intraorbital gland that is present in most terrestrial vertebrates and is especially well-developed in blind subterranean mammals. Several functions have been ascribed to the mammalian HG, including pheromonal, thermoregulatory, vomeronasal photoreceptive, and orbital lubricatory roles, and it is also involved in the regulation of the circadian rhythm [19,20]. The HG is additionally characterized by the presence of mast cells, macrophages, and immunocompetent cells. Several studies have noted the presence of macrophages in HG [21,22,23]; the HG in hamsters (*Mesocricetus auratus*) exhibits a marked sexual dimorphism in terms of cell types, and it exhibits dimorphic features in porphyrins, fatty acids, indoleamines, and somatostatin biosynthesis that could be modified by sex steroids [19]. Androgens regulate these sex differences [24,25,26,27,28,29,30,31,32]. Ovarian steroids are additionally necessary to maintain the structure and activity of the female HG, and androgen administration results in the masculinization of the HG [33,34,35].

Despite the fact that the hamster HG has been considered to be a localization site of macrophages, the role of transcriptional factor MafB in the hamster HG remains unclear. The purpose of this study was to isolate, clone, and sequence the complete cDNA encoding MafB in the hamster HG and investigate its expression in adult hamsters. Therefore, the objective of this study was to identify the participation of MafB in HG macrophages and different tissues. If the HG contains macrophages, it could be hypothesized that this intraocular tissue expresses the MafB transcript.

## 2. Material and Methods

### 2.1. Hamsters

Thirty-five adult hamsters (*Mesocricetus auratus*: 150–200 g; 8 months old; morphological analysis revealing well-defined fur; necropsy revealing no tissue pathologies, bacteria, or parasites) were housed under 12 h/12 h light/dark cycle conditions and were watered and fed ad libitum (male = 15 and female = 20). The hamsters were obtained by the Universidad Autónoma Metropolitana-Xochimilco (UAM-X; México City, México) from an animal care and use facility (code number AUT-B-C-0215-016). The animals were divided into the following seven groups: intact males (*n* = 5), males castrated seven days ago (*n* = 5), and males castrated seven days ago and intra-muscularly injected (10 µg) daily with dihydrotestosterone (DHT) or the vehicle alone (50 μL corn oil) (*n* = 5). The four stages of the estrous cycle [proestrus (P; *n* = 5), estrus (E; *n* = 5), metestrus (M; *n* = 5), and diestrus (D; *n* = 5)] were determined in female animals using vaginal smears; groups of 20 animals in each stage were defined. The male hamsters were anesthetized with ketamine/xylazine (80 mg/kg:8 mg/kg, intramuscularly) prior to gonadectomy and decapitated 24 h after the last injection. A 1.5 cm incision was made at the scrotum, and the testes were exposed, ligated, and extracted, and then the wound was sutured closed. Several tissues were immediately removed, frozen on dry ice, and stored at −70 °C until the experiments were performed [30,31,32,35]. In this study, the HGs of the males and females under different endocrine conditions were used to determine the sex steroid-dependent effect on MafB expression. The animals’ HG, lungs, liver, epididymis, heart, uterus, brain, hypothalamus, guts, spleen, testes, ovaries, adrenals, and pancreas were collected. The Ethics Committee for Research in Animals at Instituto Nacional de Ciencias Médicas y Nutrición Salvador Zubirán (INCMNSZ, BRE-1930-18-19-1) approved the research for the care and use of animals. All methods are reported in accordance with Animal Research: Reporting of In Vivo Experiments (ARRIVE) 2.0 guidelines for the reporting of animal experiments.

### 2.2. RNA Isolation and cDNA Synthesis

TRIzol reagent (Invitrogen, Carlsbad, CA, USA) was used, as recommended by the supplier, to extract total RNA. The concentration and purity of the total RNA was evaluated using a Beckmann (DU 650, Fullerton, CA, USA) spectrophotometer (at 260/280 nm, optical density: 1.8). The integrity of the isolated RNA was assessed by running the RNA samples directly on denaturing formaldehyde-agarose gel (1.2%) stained with ethidium bromide; the RNA was verified by the presence of large and small ribosomal RNA. The first-strand cDNA was synthesized from (1 μg) total RNA using a Transcriptor First Strand cDNA Synthesis kit (Roche Diagnostics, Indianapolis, IN, USA) and a Maxima First Strand cDNA Synthesis kit for RT-qPCR (ThermoScientific, Vilnius, Lithuania), according to the manufacturer’s guidelines. All the cDNA samples were stored at −20 °C until analysis.

### 2.3. Molecular Cloning

Two primers (forward 5′-gactcatctcgaggacctgta-3′ and reverse 5′-cgttctccaggtgatgtttct-3′) were designed to obtain via polymerase chain reaction (PCR) a partial sequence of hamster MafB cDNA. Primers were designed from the nucleotide sequences of mouse (NM_010658.3), rat (NM_019316.2), and human (NM_005461.5) MafB cDNA. The PCR fragment was sequenced. Based on the partial cDNA sequence obtained using PCR sequencing, two gene-specific primers (reverse 5′-cctcagggttcatctgctggtagtt-3′ for 5′-end and forward 5′-cccagtcgtgcaggtataaaacgcgt-3′ for 3′-end) were designed to amplify the 5′-end and 3′-end of MafB using a SMART Rapid Amplification of cDNA Ends (RACE) kit (Clontech, Mountain View, CA, USA), in accordance with the manufacturer’s guidelines. These fragments were sequenced, and two specific primers (forward 5′-cgttggctccgcgagt-3′ and reverse 5′-acaggacagggagtcagg-3′) were synthesized for amplifying complete MafB cDNA. The amplified PCR product was purified using electroelution/Amicon ultra-4 10 k centrifugal filter devices (Merck Millipore Ltd., Tullagreen, Carrigtwohill, Co Cork IRL) and cloned using a TOPO-TA Cloning Kit for Sequencing (Invitrogen/ThermoFisher, Waltham, MA, USA). Plasmid cDNA was isolated using a GenElute Five-Minute Plasmid Miniprep kit (Sigma-Aldrich, St. Louis, MO, USA) and a PureYield Plasmid Maxiprep kit (Promega, Woods Hollow, Madison, WI, USA), according to the manufacturer’s guidelines [30,31,32,35].

### 2.4. Sequencing and Bioinformatic Analysis

The nucleotide sequence of full-length cDNA and partial fragments of hamster MafB were determined using a BigDye Terminator v3.1 Cycle Sequencing kit (Applied Biosystems, Austin, TX, USA). The thermal cycling conditions included 1 min at 96 °C, followed by 35 cycles at 96 °C for 10 s, 50 °C for 5 s, and 60 °C for 4 min (Veriti 96 well Thermal Cycler, Applied Biosystems, Marsiling, Singapore). The resulting material was then purified using a BigDye XTerminator Purification kit (Applied Biosystems, Austin, TX, USA) and run on an ABI-PRISM 310 genetic analyzer Applied Biosystems, Foster City, CA, USA), following the manufacturer’s guidelines. The electrophoresis conditions were as follows: temperature: 50 °C; injection voltage: 15 kV; injection time: 5–7 s; 5–8 µA; the run module was KB_310POP6_BDTv3_36Rapid. The sequencing reactions were performed in the forward and reverse directions in two independent experiments. The amino acid MafB sequence was determined using an Expert Protein Analysis System (https://web.expasy.org/translate/, accessed on 3 July 2023) [36]. The molecular weight and theoretical isoelectric point of the hamster MafB were predicted using ExPASy ProtParam (https://www.web.expasy.org/protparam/, accessed on 3 July 2023) [36]. The multiple sequence alignments with other mammalian MafB proteins were determined using the CLUSTALW program (http://www.genome.jp/tools/clustalw/, accessed on 3 July 2023) [37], and the identity between the amino acid MafB sequences was performed using the Basic Local Alignment Search Tool (BLAST) program (https://blast.ncbi.nlm.nih.gov/Blast.cgi, accessed on 3 July 2023) [38]. The construction of a protein–protein interaction network was carried out via GeneMANIA (https://genemania.org/, accessed on 3 July 2023) [39]. The subcellular localization of hamster MafB was identified using DeepLoc-1.0, a eukaryotic protein subcellular localization predictor (http://www.cbs.dtu.dk/services/DeepLoc/, accessed on 3 July 2023) [40].

### 2.5. MafB Phylogenetic Tree and Three-Dimensional Structure Prediction

One hundred and twenty-five amino acid sequences of mammalian MafB were downloaded from the NCBI (https://www.ncbi.nlm.nih.gov/protein, accessed on 10 July 2023) [38] database and aligned with the multiple sequence alignment tool CLUSTALW (https://www.genome.jp/tools-bin/clustalw, accessed on 10 July 2023) [37]. The multiple alignment formats were obtained in FASTA file format using the protein database for MafB (https://www.ncbi.nlm.nih.gov/protein/, accessed on 10 July 2023) [38]. The multiple sequence alignment of the predicted/modeled and validated/reviewed MafB proteins from mammals was used to construct a phylogenetic tree. To do so, Molecular Evolutionary Genetics Analysis (MEGA) X software (https://www.megasoftware.net/, accessed on 17 July 2023) [41] was used. The evolutionary history was inferred using the maximum likelihood method and a Jones–Taylor–Thornton (JTT) matrix-based model. The tree with the highest log likelihood (−10,399.30) is shown in the results. We used the Robetta software package (http://robetta.bakerlab.org, accessed on 24 July 2023) [42] to determine the 3D structure of hamster MafB. We additionally visualized the 3D structure using PyMOL version 2.3 (http://www.pymol.org/, accessed on 24 July 2023) [43].

### 2.6. Gene Expression Analysis

The isolation of total RNA was carried out using TRIzol reagent (Invitrogen, Carlsbad, CA, USA), according to the manufacturer’s guidelines. A total of 2 µg of total RNA was used for reverse transcription with the Maxima First Strand cDNA Synthesis kit for qPCR (ThermoScientific, Vilnius, Lithuania). Reverse transcription was performed according to the manufacturer’s guidelines. qPCR was carried out in a LightCycler 2.0 system from Roche (Roche Diagnostic, GmBH, Rotkreuz, Switzerland) with LightCycler TaqMan Master Mix and pre-validated TaqMan hydrolysis probes (Roche Diagnostics, Mannheim, Germany). The relative level of MafB mRNA (sense: 5′-acgctgcagagcttcgac-3′ and antisense 5′-ctgggtacccgtggtgag-3′, 82 bp) was normalized based on the level of hamster β-actin mRNA (sense: 5′-agctatgagctgcctgatgg-3′ and antisense: 5′-caggaaggaaggctggaaa-3′; 82 bp). Transcripts of MafB and β-actin were detected using the universal fluorogenic probes #77 (04689003001) and #9 (04685075001), respectively. The cycling conditions were 95 °C for 10 min, 40 cycles of amplification at 95 °C for 10 s, 60 °C for 30 s, 72 °C for 1 s, and a final cycle of cooling at 40 °C. The qPCR data were analyzed using the relative quantification method provided by LightCycler software 4.1, and they are expressed in arbitrary mRNA units as the mean ± standard deviation (SD) of five biological independent replicates for each group. Relative gene expressions were quantified using the 2^−ΔΔCt^ calculation method. In order to gain insights into whether the hamster HG exhibited differential expression patterns as a function of sex steroids and sex condition, an MafB mRNA analysis of intact and castrated males was performed.

### 2.7. Statistical Analysis

Differences in MafB mRNA levels were assessed using one-way ANOVA. A *p* value less than 0.05 was considered to indicate statistical significance.

## 3. Results

### 3.1. cDNA Cloning of Hamster MafB

**Figure 1:**
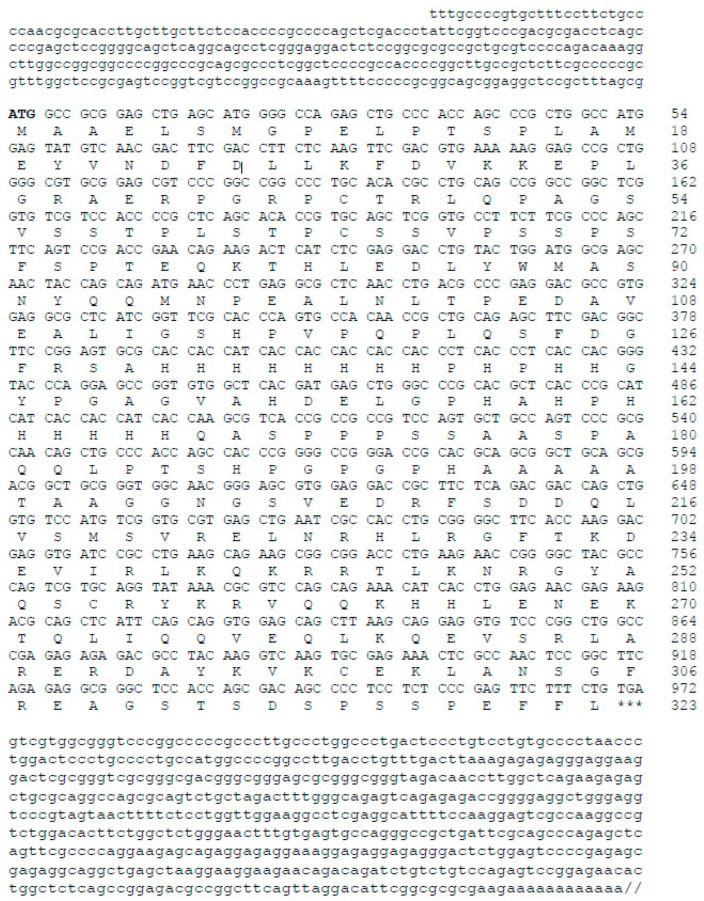
Nucleotide and deduced amino acid sequence of hamster MafB. The numbers on the right indicate the positions of the nucleotides and amino acids. The start codon ATG is indicated in bold and the stop codon TGA is indicated with three asterisks. The MafB cDNA contained a 969-bp ORF that encoded a 323-amino acid polypeptide. The nucleotide sequences of the 5′ and 3′ ends of the MafB cDNA consisted of 308 and 1789 bp, respectively. The diagonal lines represent a 3′-UTR of 1203 bp. The GenBank accession number of hamster MafB is MZ215994.

DNA sequencing analysis revealed that the isolated and cloned cDNA of hamster MafB consisted of 3066 base pairs (bp), with a 5′-untranslated region (5′-UTR) of 308 bp and a 3′-UTR of 1789 bp. The isolated cDNA clone contained an open reading frame (ORF) of 969 bp encoding 323 amino acids (Figure 1). The nucleotide and deduced amino acid sequences were submitted to the GenBank database (accession number MZ215994).

### 3.2. Characterization of MafB Protein from Hamsters

**Figure 2:**
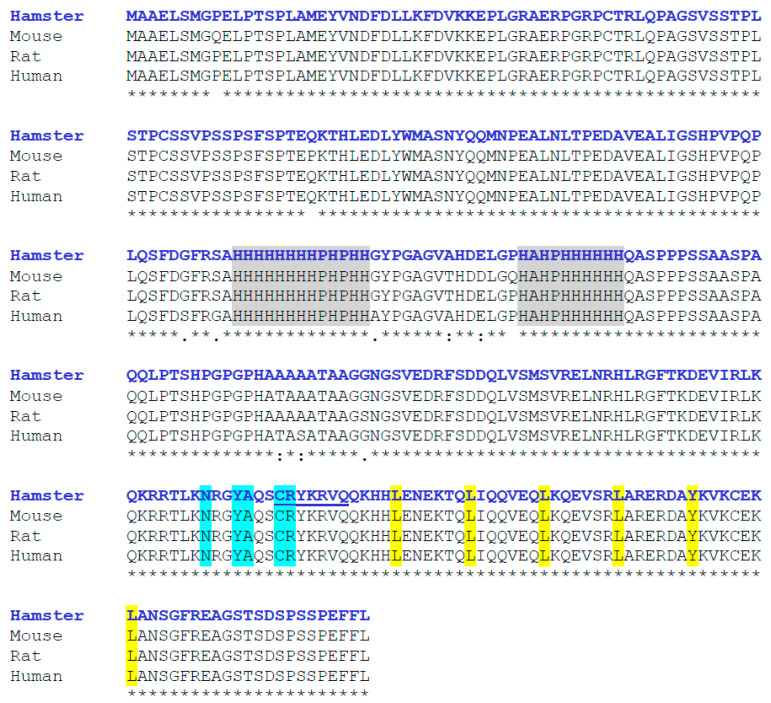
Multiple alignment of amino acid sequences of hamster MafB with those of other mammalian species (*Mus musculus*, *Rattus norvegicus*, and *Homo sapiens*). Asterisks indicate amino acids consistent across all four sequences. The two histidine-rich boxes are indicated in gray. At the COOH-terminal end, the DNA-binding domain is shown in blue, the nuclear localization signal is underlined, and the bZIP domain appears in yellow. Sequence alignment was performed using ClustalW.

The estimated molecular weight of the deduced peptide was 35,731.75 Da, and the theoretical isoelectric point (pI) was 7.17. Using ClustalW 2.1 and BLAST (https://blast.ncbi.nlm.nih.gov/Blast.cgi, accessed on 3 July 2023) softwares, hamster MafB was characterized by a high level of identity (98–99%) with other MafB amino acid sequences from mice (NP_034788.1), rats (NP_062189.1), and humans (NP_005452.2). The protein sequence exhibited all the features of MafB proteins, including two histidine-rich boxes (131–143 and 158–167 residues) localized in the central region of the sequence. The deduced amino acid sequence predicted a bZIP domain (L_266_, L_273_, L_280_, L_287_, Y_294_, and L_301_) between amino acids 238 and 301. I identified a DNA-binding domain (N_248_XXY_251_A_252_XXC_255_R_256_) and a putative nuclear localization signal (likelihood 0.99; C_255_, R_256_, Y_257_, K_258_, R_259_, V_260_, and Q_261_) at its COOH-terminal end (Figure 2).

**Figure 3:**
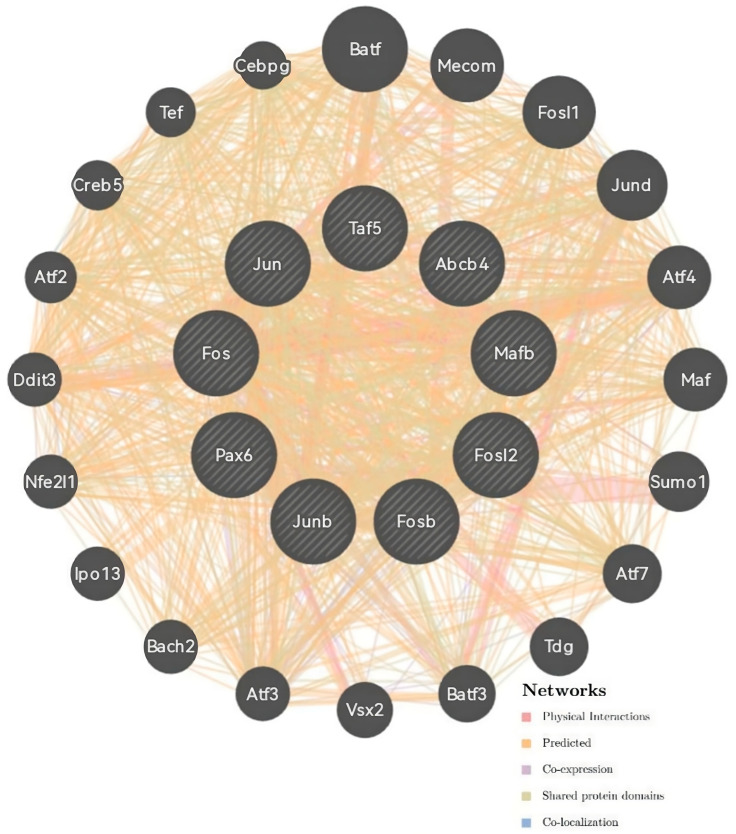
Correlation analysis of co-expression predictions and the physical interaction network of the *MafB* gene by GeneMania. The top nine genes displaying the greatest correlations with MafB included *Abcb4*, *Nfe2l1*, *Fosl2*, *Fosb*, *Junb*, *Jun*, *Fos*, *Taf5*, and *Pax6*.

Next, protein and genetic interactions, pathways, co-expression, and co-localization and protein domain similarity were analyzed using the GeneMANIA server (Figure 3). Co-expression predictions were identified with ATP-binding cassette, sub-family B, member 4 (Abcb4); nuclear factor, erythroid-derived 2,-like 1 (Nfe2l1), FOS-like antigen 2 (Fosl2); FBJ osteosarcoma oncogene B (Fosb); JunB proto-oncogene (Junb); and Jun proto-oncogene (Jun). Physical interactions were predicted using Fos proto-oncogene (Fos), TATA-box binding protein associated factor 5 (Taf5), and paired box 6 (Pax6).

### 3.3. Phylogenetic Analysis of MafB Proteins in Mammals and Three-Dimensional Structure of Hamster MafB Protein

**Figure 4:**
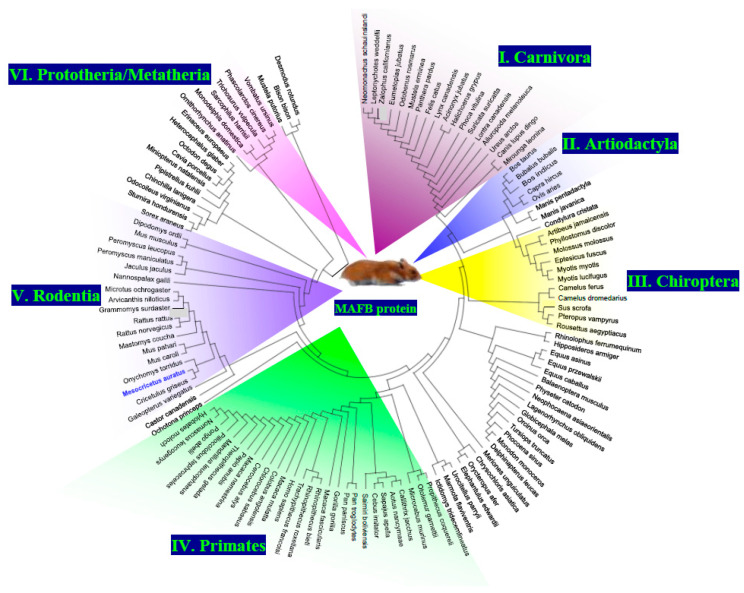
Phylogenetic analysis of the MafB protein of hamster (blue) and other mammalian species. The phylogenetic tree was assembled using MEGA X and was constructed using the maximum likelihood method and a JTT matrix-based model. This evolutionary analysis involved 126 amino acid sequences, as follows: NP_034788.1 (*Mus musculus*), NP_005452.2 (*Homo sapiens*), NP_062189.1 (*Rattus norvegicus*), XP_001101624.3 (*Macaca mulatta*), XP_002692508.1 (*Bos Taurus*), XP_525325.3 (*Pan troglodytes*), XP_012908766.1 (*Mustela putorius furo*), XP_023482332.1 (*Equus caballus*), XP_002747609.1 (*Callithrix jacchus*), XP_007474404.1 (*Monodelphis domestica*), XP_031809881.1 (*Sarcophilus harrisii*), XP_020846473.1 (*Phascolarctos cinereus*), XP_036607718.1 (*Trichosurus vulpecula*), XP_027723123.1 (*Vombatus ursinus*), XP_004585865.1 (*Ochotona princeps*), XP_012866530.1 (*Dipodomys ordii*), XP_020038332.1 (*Castor Canadensis*), XP_021005512.1 (*Mus caroli*), XP_021048660.1 (*Mus pahari*), XP_015854992.1 (*Peromyscus maniculatus bairdii*), XP_004669340.1 (*Jaculus jaculus*), XP_021500039.1 (*Meriones unguiculatus*), XP_032760554.1 (*Rattus rattus*), XP_028642582.1 (*Grammomys surdaster*), XP_031228459.1 (*Mastomys coucha*), XP_034352409.1 (*Arvicanthis niloticus*), XP_008847806.1 (*Nannospalax galili*), XP_027277031.1 (*Cricetulus griseus*), XP_028724218.1 (*Peromyscus leucopus*), XP_036042220.1 (*Onychomys torridus*), XP_005363051.1 (*Microtus ochrogaster*), XP_005002829.1 (*Cavia porcellus*), XP_005392579.1 (*Chinchilla lanigera*), XP_004630965.1 (*Octodon degus*), XP_004874085.1 (*Heterocephalus glaber*), XP_005329891.1 (*Ictidomys tridecemlineatus*), XP_026239080.1 (*Urocitellus parryii*), XP_027801177.1 (*Marmota flaviventris*), XP_026925892.1 (*Acinonyx jubatus*), XP_030165456.1 (*Lynx canadensis*), XP_023107022.1 (*Felis catus*), XP_019314045.1 (*Panthera pardus*), XP_025325854.1 (*Canis lupus dingo*), XP_026359290.1 (*Ursus arctos horribilis*), XP_032694433.1 (*Lontra canadensis*), XP_032206561.1 (*Mustela erminea*), XP_004412027.1 (*Odobenus rosmarus divergens*), XP_029772639.1 (*Suricata suricatta*), XP_019654468.2 (*Ailuropoda melanoleuca*), XP_027977768.1 (*Eumetopias jubatus*), XP_027479414.1 (*Zalophus californianus*), XP_021544629.1 (*Neomonachus schauinslandi*), XP_035977278.1 (*Halichoerus grypus*), XP_006746707.1 (*Leptonychotes weddellii*), XP_034862207.1 (*Mirounga leonina*), XP_032275900.1 (*Phoca vitulina*), XP_020725392.1 (*Odocoileus virginianus texanus*), XP_010827540.1 (*Bison bison bison*), XP_027414344.1 (*Bos indicus x Bos taurus*), XP_006065979.1 (*Bubalus bubalis*), XP_017913355.1 (*Capra hircus*), XP_012044596.3 (*Ovis aries*), XP_013840831.2 (*Sus scrofa*), XP_032318191.1 (*Camelus ferus*), XP_031289675.1 (*Camelus dromedarius*), XP_024598990.1 (*Neophocaena asiaeorientalis asiaeorientalis*), XP_026934105.1 (*Lagenorhynchus obliquidens*), XP_030699450.1 (*Globicephala melas*), XP_004272898.1 (*Orcinus orca*), XP_004311902.1 (*Tursiops truncatus*), XP_032462789.1 (*Phocoena sinus*), XP_029077669.1 (*Monodon monoceros*), XP_022448116.2 (*Delphinapterus leucas*), XP_028355533.1 (*Physeter catodon*), XP_036681764.1 (*Balaenoptera musculus*), XP_007521706.1 (*Erinaceus europaeus*), XP_004612547.1 (*Sorex araneus*), XP_004687441.1 (*Condylura cristata*), XP_011364910.1 (*Pteropus vampyrus*), XP_015993032.1 (*Rousettus aegyptiacus*), XP_032950919.1 (*Rhinolophus ferrumequinum*), XP_036899957.1 (*Sturnira hondurensis*), XP_037020425.1 (*Artibeus jamaicensis*), XP_024415040.1 (*Desmodus rotundus*). XP_028380257.1 (*Phyllostomus discolor*), XP_016074787.1 (*Miniopterus natalensis*), XP_019492655.1 (*Hipposideros armiger*), XP_036274169.1 (*Pipistrellus kuhlii*), XP_008156672.1 (*Eptesicus fuscus*), XP_036177934.1 (*Myotis myotis*), XP_006085239.1 (*Myotis lucifugus*), XP_036101794.1 (*Molossus molossus*), XP_014701439.1 (*Equus asinus*), XP_008510758.1 (*Equus przewalskii*), XP_036758180.1 (*Manis pentadactyla*), XP_017504927.2 (*Manis javanica*), XP_008587889.1 (*Galeopterus variegatus*), XP_011784437.1 (*Colobus angolensis palliatus*), XP_008015551.1 (*Chlorocebus sabaeus*), XP_011921032.1 (*Cercocebus atys*), NP_001306451.1 (*Macaca fascicularis*), XP_011764992.1 (*Macaca nemestrina*), XP_003904790.1 (*Papio anubis*), XP_025255497.1 (*Theropithecus gelada*), XP_011830354.1 (*Mandrillus leucophaeus*), XP_033042310.1 (*Trachypithecus francoisi*), XP_017714609.1 (*Rhinopithecus bieti*), XP_010372973.1 (*Rhinopithecus roxellana*), XP_023083683.1 (*Piliocolobus tephrosceles*), XP_004062201.1 (*Gorilla gorilla gorilla*), XP_003825940.1 (*Pan paniscus*), XP_002830352.1 (*Pongo abelii*), XP_003253627.1 (*Nomascus leucogenys*), XP_031998418.1 (*Hylobates moloch*), XP_003936434.1 (*Saimiri boliviensis boliviensis*), XP_032150831.1 (*Sapajus apella*), XP_017367441.1 (*Cebus imitator*), XP_012310439.2 (*Aotus nancymaae*), XP_012507521.1 (*Propithecus coquereli*), XP_012610703.1 (*Microcebus murinus*). XP_003787643.1 (*Otolemur garnettii*), XP_007932858.1 (*Orycteropus afer afer*), XP_006881591.1 (*Elephantulus edwardii*), XP_006839358.1 (*Chrysochloris asiatica*), and XP_028926613.1 (*Ornithorhynchus anatinus*).

**Figure 5:**
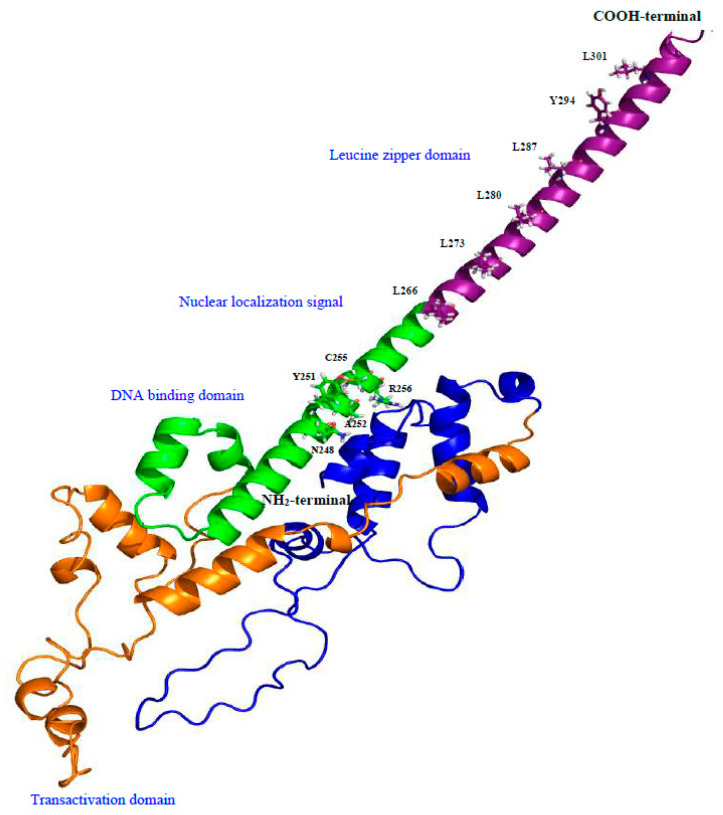
Three-dimensional protein model generated using Robetta software. The domain organization of hamster MafB protein is indicated in different colors, and each domain is labeled with corresponding amino acids. The NH_2_-terminal end is colored blue and orange, DNA-binding domain and nuclear localization signal is colored in green, and the COOH-terminal end is colored purple.

The results of the phylogenetic tree revealed that all the MafB proteins could be separated into six different groups (groups I–VI; Figure 4). The 3D structure of MafB was estimated using the Robetta software package (http://robetta.bakerlab.org, accessed on 24 July 2023) and visualized with PyMOL. This model, based on the hamster MafB protein sequence (Figure 5), revealed several domains as follows: a leucine zipper domain, a nuclear localization signal, a DNA-binding domain, and a transactivation domain.

### 3.4. MafB Gene Expression in Hamsters

**Figure 6:**
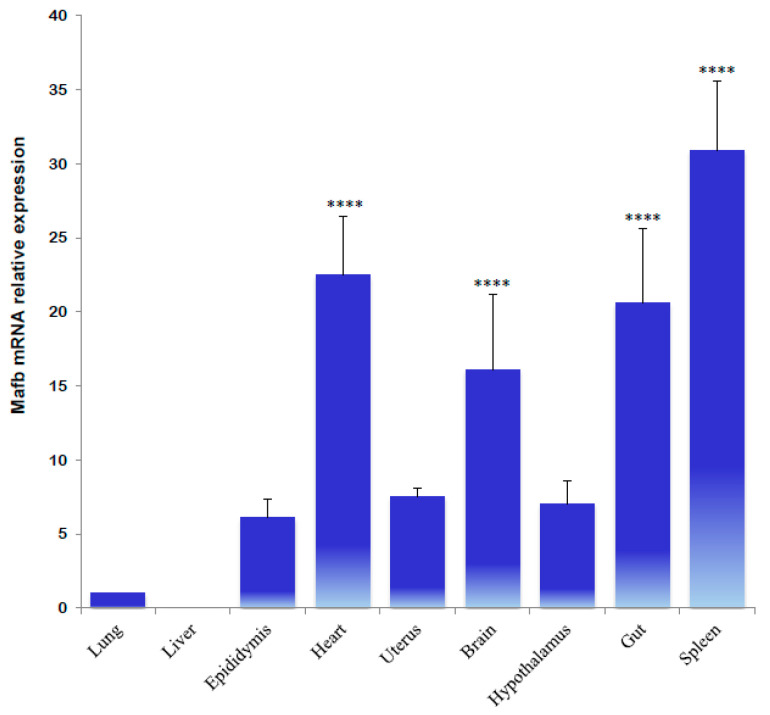
Tissue distribution of hamster MafB transcript expression determined using qPCR. The data refer to Mafb mRNA relative expression and are provided as means (bars) ± SD. **** indicates *p* < 0.0001.

Quantitative real-time polymerase chain reaction (qPCR) analysis revealed multiple variations in each hamster tissue and sex condition. Initially, the MafB transcript was characterized by a broad distribution in adult hamster tissues. The most abundant transcripts were identified in the spleen, gut, heart, and brain; the least abundant transcripts were identified in the liver, lungs, epididymis, uterus, and hypothalamus (Figure 6).

**Figure 7:**
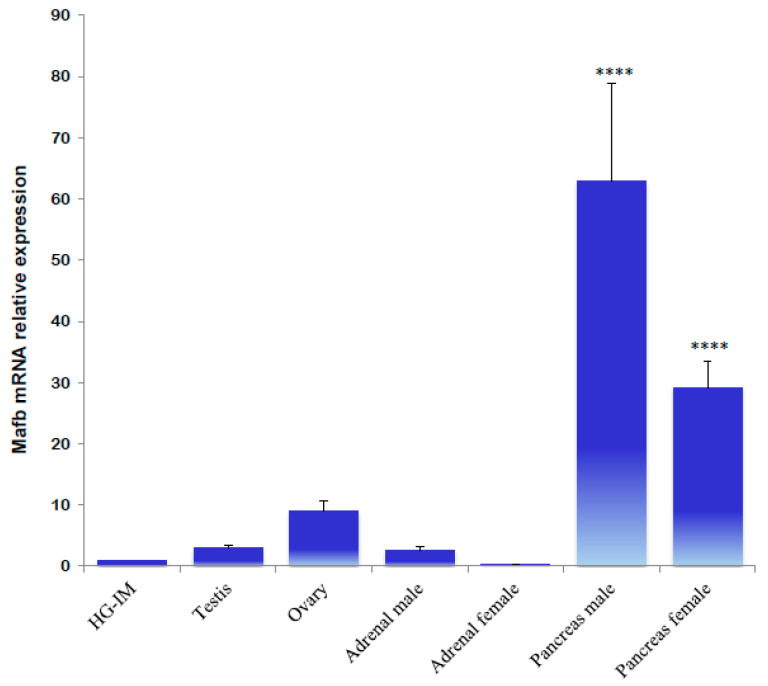
Gene expression analysis of hamster MafB transcript in endocrine tissues using qPCR. The assessed tissues included a HG of an intact male (HG-IM). The relative expression levels of the hamster *MafB* gene were normalized by the expression of the *β-actin* gene. The values represent the mean (bars) ± SD (*n* = 5 biological independent replicates). **** indicate *p* < 0.0001.

qPCR expression values of the *MafB* gene in endocrine tissues revealed that all samples are differentially expressed. Pancreatic tissue from male and female hamsters exhibited the highest average levels of *MafB* gene expression; the transcripts in male and female (metestrus) adrenals were significantly lower than in the testes and ovaries (Figure 7).

**Figure 8:**
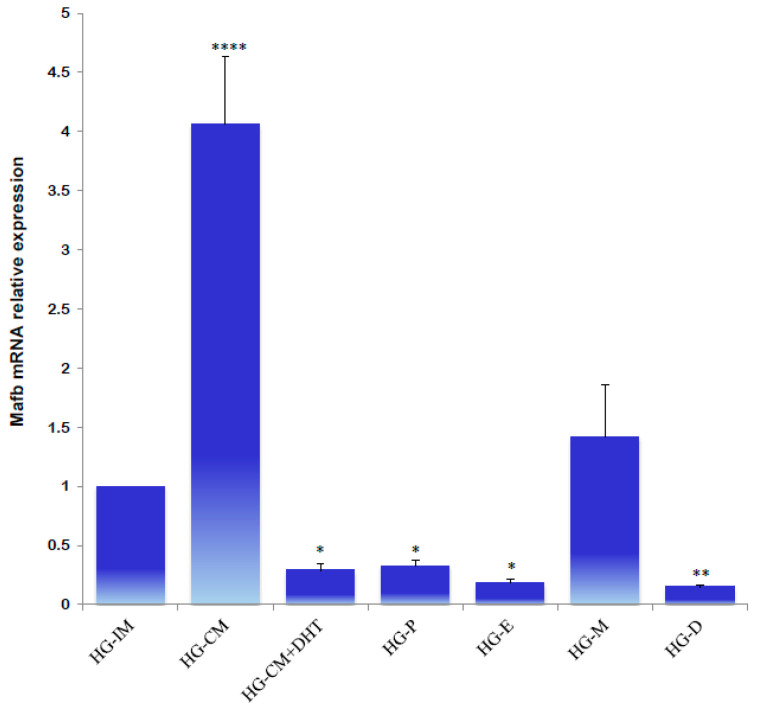
Gene expression levels of the MafB transcript from hamster HG determined using qPCR. Data from an intact male (HG-IM), a castrated male (HG-CM), a castrated male that received DHT (HG-CM + DHT), a female in proestrus (HG-P), a female in estrus (HG-E), a female in metestrus (HG-M), and a female in diestrus (HG-D) are shown. The relative expression levels of the *MafB* gene were calculated relative to the expression of the *β-actin* gene. All the data are expressed as means (bars) ± SD (*n* = 5 biological independent replicates). ****, ** and * indicate *p* < 0.0001, *p* < 0.01, and *p* < 0.05, respectively.

A significantly higher relative expression of male MafB was detected in the castrated males; the administration of DHT reestablished the transcript levels. In the HG of female hamsters, similar expression levels were determined in different phases of the estrous cycle. A slightly higher expression was noted during metestrus; however, MafB mRNA did not exhibit a sexually dimorphic expression pattern (Figure 8).

**Figure 9:**
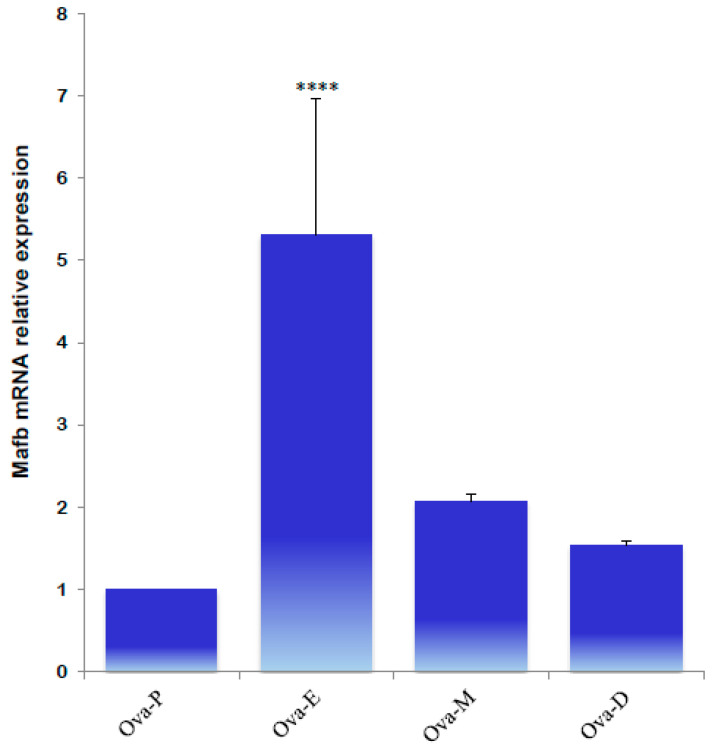
qPCR quantification of MafB mRNA expression levels from hamster ovaries in proestrus (Ova-P), estrus (Ova-E), metestrus (Ova-M), and diestrus (Ova-D) females. The gene of interest was normalized to the reference gene (*β-actin*), and the relative expression levels were compared using the relative ΔΔC_t_ method. The data are presented as means (bars) ± SD (*n* = 5 biological independent replicates). **** indicates *p* < 0.0001.

**Figure 10:**
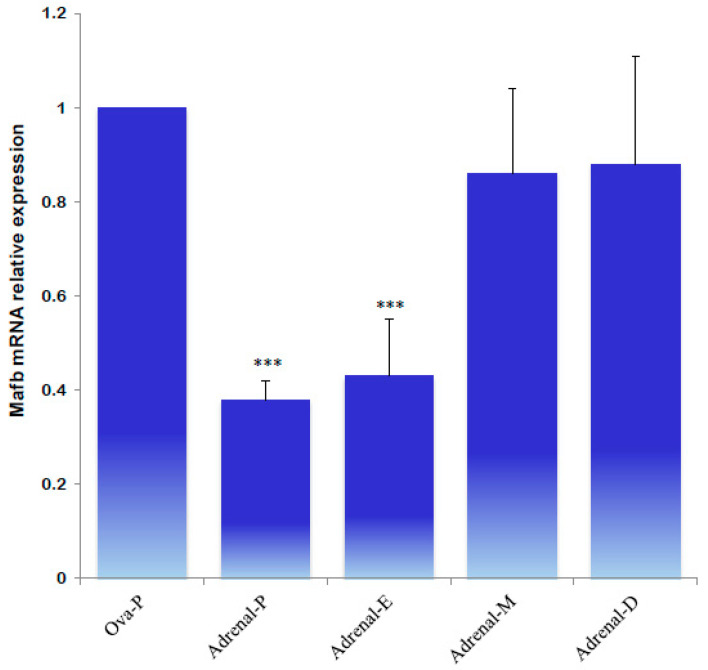
qPCR analysis of MafB transcript expression levels from hamster adrenals in proestrus (P), estrus (E), metestrus (M), and diestrus (D) females. The assessed tissues included an ovary of an intact female in proestrus (Ova-P). *β-actin* was used as a reference gene. The data are presented as means (bars) ± SD (*n* = 5 biological independent replicates). *** indicate *p* < 0.001.

Next, the expression profiles of MafB were characterized using qPCR to identify MafB expression in endocrine tissues during the animals’ estrous cycle. Ovarian tissue obtained during estrus “exhibited high relative expression” of the MafB transcript; mRNA expression was constant during the other phases (proestrus, metestrus, and diestrus) (Figure 9). Endocrine *MafB* gene expression was compared to *MafB* gene expression in the adrenals during the animals’ estrous cycle, and no significant changes in MafB relative expression levels were observed. (Figure 10).

**Figure 11:**
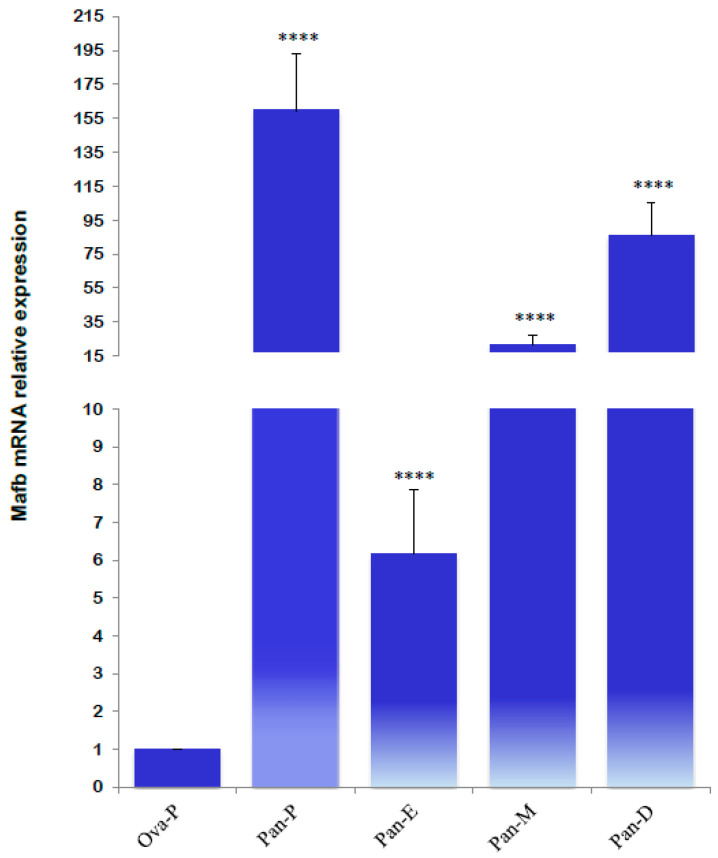
Relative mRNA expression levels of MafB transcript from hamster pancreas in proestrus (Pan-P), estrus (Pan-E), metestrus (Pan-M), and diestrus (Pan-D) females. The assessed tissues included an ovary of an intact female in proestrus (Ova-P). *β-actin* was used as a reference gene. The relative expression level was calculated using the ΔΔC_t_ method. Each value of MafB mRNA represents means (bars) ± SD (*n* = 5 biological independent replicates). **** indicates *p* < 0.0001.

The mRNA gene relative expression profiles revealed significantly higher MafB transcript levels during metestrus, diestrus, and proestrus. The lowest MafB relative expression was noted in estrus; significantly higher expression levels were detected in the proestrus pancreas (Figure 11).

## 4. Discussion

The transcription factor MafB is expressed in pancreatic α cells, and it plays a role in embryonic urethral formation [9,16,44,45,46], MafB is also expressed in macrophages and regulates their differentiation [47]. In rodents, the HG is an important site of macrophage localization [21]. Even so, the expression and regulation of the *MafB* gene in the hamster HG remains largely unknown. The results revealed the amplification of a cDNA fragment of 3066 bp covering the entire coding region. The ORF was composed of 969 bp, which is consistent with sequences from other mammalian species [4,5,48], and encodes a deduced protein of 323 amino acid residues of 35731.75 Da. The hamster peptide possessed the highly conserved motifs of MafB, such as a DNA-binding domain, a nuclear signal domain, and a leucine zipper domain. A phylogenetic tree additionally revealed that hamster MafB has a similar evolutionary line; hamster MafB proteins are closely related to those of the Rodentia order. The proteins were characterized by the orders Carnivora (I), Artiodactyla (II), Chiroptera (III), Primates (IV), Rodentia (V), and a small group of Prototheria/Metatheria (VI). In some mammalian groups, such as the order Chiroptera, the set of MafB proteins was interrupted by proteins of other orders. The hamster MafB protein was included in the Rodentia group. The amino acid sequence of mature hamster MafB peptide exhibited rather high identities and highly conserved structural features. These results suggest that the physiological functions of this peptide are conserved among mammals. In summary, these results pertaining to molecular properties, sequence similarity, conserved domains, phylogenetic relationships, and structural characterization support the assertion that the *MafB* gene identified in this study is hamster MafB.

The MAfB transcript has been ubiquitously detected in all human tissues examined using Northern blot assays; however, the transcript is expressed predominantly in bone, bone marrow, skeletal muscle, and the heart. Northern blot assays also revealed expression in the spleen, brain, and pancreas [4]. This study used qPCR assays to further identify strong expression in the epididymis, uterus, and hypothalamus. The pronounced expression of the MafB transcription factor in these tissues suggests that this transcription factor participates in cellular maintenance and differentiation, specifically in macrophages, in adult hamsters. The findings suggested that MafB functioning may be necessary for macrophage differentiation in spleen cells. On the other hand, MafB mRNA has been reported to exhibit significantly high mRNA levels in goldfish (*Carassius auratus* L.) spleen tissue [49]. However, relatively little is known about the relationship between MafB and splenocytes.

The MafB transcript was principally detected in the pancreas of both male and female hamsters. A sexually dimorphic or estrous cycle-dependent expression of MafB was not evident in other endocrine tissues (e.g., the testes, ovaries, and adrenals). However, moderate sexual dimorphism was observed in the gonads, and MafB relative expression was increased in the animals’ ovaries. Numerous reports have identified MafB transcription factor to be present in human and mouse pancreatic *β* cells [7,50,51,52,53]. In islet β cells, MafB activity regulates many genes essential to glucose sensing and insulin secretion in a cooperative and sequential manner. In addition, MafB was present in both insulin- and glucagon-producing cells during development, with expression only restricted to α cells soon after birth [7]. Furthermore, MafB is required for insulin and glucagon transcription in developing α and β cells [7,51]. In addition to identifying MafB mRNA expression in the hamster pancreas, this study also detected sexually dimorphic and sex steroid-dependent expression. These results suggest that sex steroids (likely estrogen and/or progesterone) might regulate MafB expression and therefore control insulin and glucose secretion during the phases of the estrous cycle. Future studies can contribute to furthering our understanding of how the steroid-receptor complex regulates the expression of the *MafB* gene in pancreatic tissue in hamsters.

Finally, the expression of MafB transcription factor has been observed in various types of cells. In chicks, the overexpression of MafB in transformed myeloblasts stimulates the rapid formation of macrophages [11]. In mice, MafB is specifically expressed in macrophages and is a critical regulator of macrophage differentiation but is also expressed in other cell types [9,47]. One study showed that nuclear receptor transcription factors are involved in the metabolic and immune activities of macrophages by regulating target genes [54]. This variability in MafB transcript expression levels might be the effect of differential gene regulation. A likely explanation is the presence of multiple mechanisms that influence the amount of transcription of the *MafB* gene, such as epigenetic modifications, genetic variants, tissue-specific transcription factors, or cellular signaling from other tissues. A somewhat sexually dimorphic expression of MafB in the HG was observed. Even so, these results indicated that MafB expression was increased due to the absence of sex steroids (i.e., male gonadectomy). These findings suggest that androgens regulate MafB expression in hamster HG. Conducting functional analyses like differential gene expression and subsequent pathway analysis will expand on the current study. Matsushita et al. [55] reported that androgens regulate MafB expression via its 3′UTR during mouse urethral masculinization. Additionally, Vilchis et al. [26] characterized and demonstrated the presence of a specific high-affinity intracellular androgen receptor (AR/NR3C4) in the HG in male hamsters. These results suggest that the androgen–AR complex regulates MafB expression. Therefore, the androgen-dependent expression of MafB might influence the phagocytic activity of macrophages in the HG. Due to the intraocular location of the HG, macrophages exist in this tissue to clear cellular and environmental debris [9]. In addition to the AR/NR3C4 transcription factor, several nuclear transcriptional factors were associated with MafB, such as Nfe2l1, Fosl2, Fosb, Junb, Jun, Fos, Taf5, and Pax6. Likewise, the gene expression profiles of other transcriptional factors, such as *Sox9* and *Dax1*, have been identified in intraocular tissue [31,56]. Although MafB protein was not examined in this intraocular tissue, the expression data suggest that MafB is likely involved in cellular maintenance and differentiation in adult hamsters.

## 5. Conclusions

In summary, this study identified and characterized the full-length cDNA sequence of MafB in hamsters. Furthermore, this study investigated the tissue-expression profiles of MafB in hamsters, suggesting a sexually dimorphic control over the expression of pancreatic/HG MafB. These results may suggest the participation of MafB in the differentiation of HG macrophages and pancreatic β cells. Future studies aimed at investigating the inflammatory, infectious, and pathological processes associated with the *MafB* gene and these tissues will be valuable.

## Data Availability

A preprint version of the manuscript is available at https://www.researchsquare.com/article/rs-3994807/v1, accessed on 7 March 2022), which was not conducted to print and publication after peer reviewing. The cDNA sequence data are available at https://www.ncbi.nlm.nih.gov/nuccore/MZ215994.1/, accessed on 21 May 2022.
